# Identification of postnatal development dependent genes and proteins in porcine epididymis

**DOI:** 10.1186/s12864-023-09827-y

**Published:** 2023-12-04

**Authors:** Shaoming Fang, Zhechen Li, Shuo Pang, Yating Gan, Xiaoning Ding, Hui peng

**Affiliations:** 1 College of Animal Science (College of Bee Science), Fujian Agriculture and Forestry University, Fuzhou, 35002 China; 2 College of Animal Science and Technology, Hainan University, Haikou, 570228 China

**Keywords:** Duroc pigs, Differentially expressed genes (DEGs), Differentially expressed proteins (DEPs), Epididymis functions, Molecular basis

## Abstract

**Background:**

The epididymis is a highly regionalized tubular organ possesses vectorial functions of sperm concentration, maturation, transport, and storage. The epididymis-expressed genes and proteins are characterized by regional and developmental dependent pattern. However, a systematic and comprehensive insight into the postnatal development dependent changes in gene and protein expressions of porcine epididymis is still lacking. Here, the RNA and protein of epididymis of Duroc pigs at different postnatal development stages were extracted by using commercial RNeasy Midi kit and extraction buffer (7 M Urea, 2 M thiourea, 3% CHAPS, and 1 mM PMSF) combined with sonication, respectively, which were further subjected to transcriptomic and proteomic profiling.

**Results:**

Transcriptome analysis indicated that 198 and 163 differentially expressed genes (DEGs) were continuously up-regulated and down-regulated along with postnatal development stage changes, respectively. Most of the up-regulated DEGs linked to functions of endoplasmic reticulum and lysosome, while the down-regulated DEGs mainly related to molecular process of extracellular matrix. Moreover, the following key genes *INSIG1*, *PGRMC1*, *NPC2*, *GBA*, *MMP2*, *MMP14*, *SFRP1*, *ELN*, *WNT-2*, *COL3A1*, and *SPARC* were highlighted. A total of 49 differentially expressed proteins (DEPs) corresponding to postnatal development stages changes were uncovered by the proteome analysis. Several key proteins ACSL3 and ACADM, VDAC1 and VDAC2, and KNG1, SERPINB1, C3, and TF implicated in fatty acid metabolism, voltage-gated ion channel assembly, and apoptotic and immune processes were emphasized. In the integrative network, the key genes and proteins formed different clusters and showed strong interactions. Additionally, *NPC2*, *COL3A1*, C3, and VDAC1 are located at the hub position in each cluster.

**Conclusions:**

The identified postnatal development dependent genes and proteins in the present study will pave the way for shedding light on the molecular basis of porcine epididymis functions and are useful for further studies on the specific regulation mechanisms responsible for epididymal sperm maturation.

**Supplementary Information:**

The online version contains supplementary material available at 10.1186/s12864-023-09827-y.

## Background

It is well known that the epididymis exerts critical roles in sperm transport, concentration, maturation, immunoprotection, and storage. Epididymis is a long and highly convoluted tube composed of three main regions: caput, corpus, and cauda, each with unique characteristics and functions [1]. Hence, specific changes in morphology and function of epididymis during different development processes could affect the epididymal transit of sperm [2]. To comprehensively understand the underlying molecular basis of epididymis functions, characterizing the gene and protein expression profiles of epididymal tissues are urgently needed.

The epididymis is divided into different regions and each comprises a considerable amount of region-specific expressed genes and proteins constitute the unique luminal microenvironment, which is important for the maturation of spermatozoa occurs during epididymis transit. Browne et al. [3] found that the differentially expressed genes (DEGs) such as aquaporin-1 (AQP1), serpin family A member 1 (SERPINA1), and fibroblast growth factor 18 (FGF18) in the human caput epididymis were enriched in processes of ion transport, response to hormone stimulus, and urogenital tract development, while DEGs such as toll-like receptor 2 (TLR2), interleukin 1 alpha (IL1A), and C-X-C motif chemokine 10 (CXCL10) were more active in defense response processes in the corpus and cauda. Beta defensin had the dual role in epididymal immunity modulation and sperm maturation, and Jelinsky et al. [4] indicated that most members of the beta defensin gene family showed highly conserved patterns of segment-dependent expression in the mouse epididymis. Inducible carbonyl reductase is an important anti-oxidase, and Yuan et al. [5] reported that its expression level greatly varied from the caput to the cauda regions of rat epididymis.

On the other hand, the variations in expression levels of epididymial genes and proteins at different postnatal developmental stages also have been found to be closely related to the functions of epididymis. Cadherins mediated the formation of the blood-epididymal barrier, and their expression levels varied dramatically at different postnatal developmental stages in the rat epididymis [6]. D-type cyclins had important implications for cell cycle control and function of the epididymis, which were expressed in a development-specific manner in the postnatal mouse epididymis [7]. Epididymal lysosomal enzymes such as β-galactosidase and α-mannosidase could modify membrane components of spermatozoa during maturation, which had greater activity in sexually immature bulls than sexually mature bulls [8]. Epididymal osteopontin exerted a role in maintaining mineral homeostasis of epididymis fluid, and its expression level gradually increased during the postnatal development of sheep [9].

Although previous studies have unraveled a considerable number of genes and proteins expressed in a region-dependent and development-dependent pattern that affect epididymis functions, most of the conclusions are drawn from the single level and the integrative analysis of transcriptome and proteome in epididymis is less conducted. In this study, tissue samples derived from the caput, corpus, and cauda epididymis of 2-, 4-, 6-, and 8-month-old Duroc pigs were mixed according to the different development stages and subjected to a systematic and comprehensive transcriptome and proteome analysis to fill gap in multi-omics studies on porcine epididymis for revealing possible new modulatory mechanisms of epididymal sperm maturation. Our results will identify some development-dependent genes and proteins involve in different physiological processes of epididymis, which should inform the understanding of molecular basis of porcine epididymal functions and provide targets for future studies to identify specific biochemical processes involved in epididymal sperm maturation.

## Results

### The characteristics of porcine epididymal transcriptome at different postnatal development stages

**Fig. 1:**
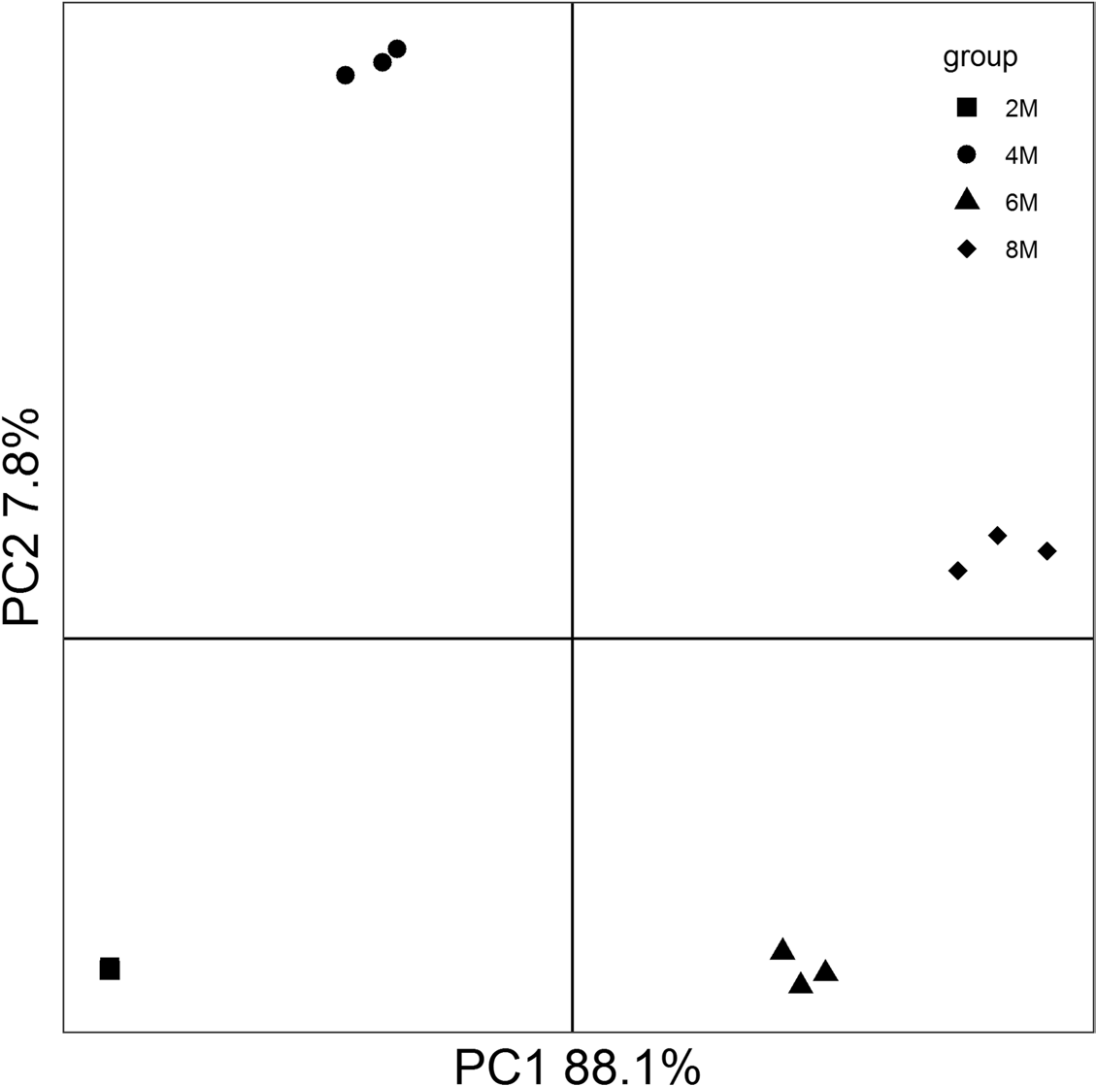
The principal component analysis (PCA) of porcine epididymal transcriptome at different postnatal development stages

A total of 21,004, 21,869, 22,391, and 22,361 genes were annotated in the 2-, 4-, 6-, and 8-month-old porcine epididymis, respectively (Fig. S1A). The Venn plot showed that 19,857 common genes were presented in the porcine epididymis at different postnatal development stages, while 188, 257, 291, 283 genes were specific to2-, 4-, 6-, and 8-month-old porcine epididymis, respectively (Fig. S1B). The PCA analysis indicated that epididymal transcriptome of the pigs formed 4 distinctive clusters according to the postnatal development stages (Fig. 1).

Subsequently, the common and specific genes were used for functional annotation analysis. As shown in Fig. S2A, the common genes were assigned to biological process, cellular component, molecular function, and KEGG pathways and each of these functional terms consisted of 16, 2, 10, and 6 categories, respectively. In the biological process, cellular process, biological regulation, localization, metabolic process, and response to stimulus were the most predominant functional categories. In the cellular component, the common genes were annotated to cellular anatomical entity and protein-containing complex. Most of the common genes in the molecular function were linked to binding, catalytic activity, transcription regulator activity, and molecular function regulator. Meanwhile, genetic information processing, metabolism, and environmental information processing were the top three functional categories in the KEGG pathways. As for the specific genes, the similar functional annotation results were obtained (Fig. S2B). Intriguingly, the gene counts of the most functional categories in the sexually mature period were generally higher than the sexually immature period.

### Identification of DEGs and functional capacity corresponding to postnatal development stages changes

**Fig. 2:**
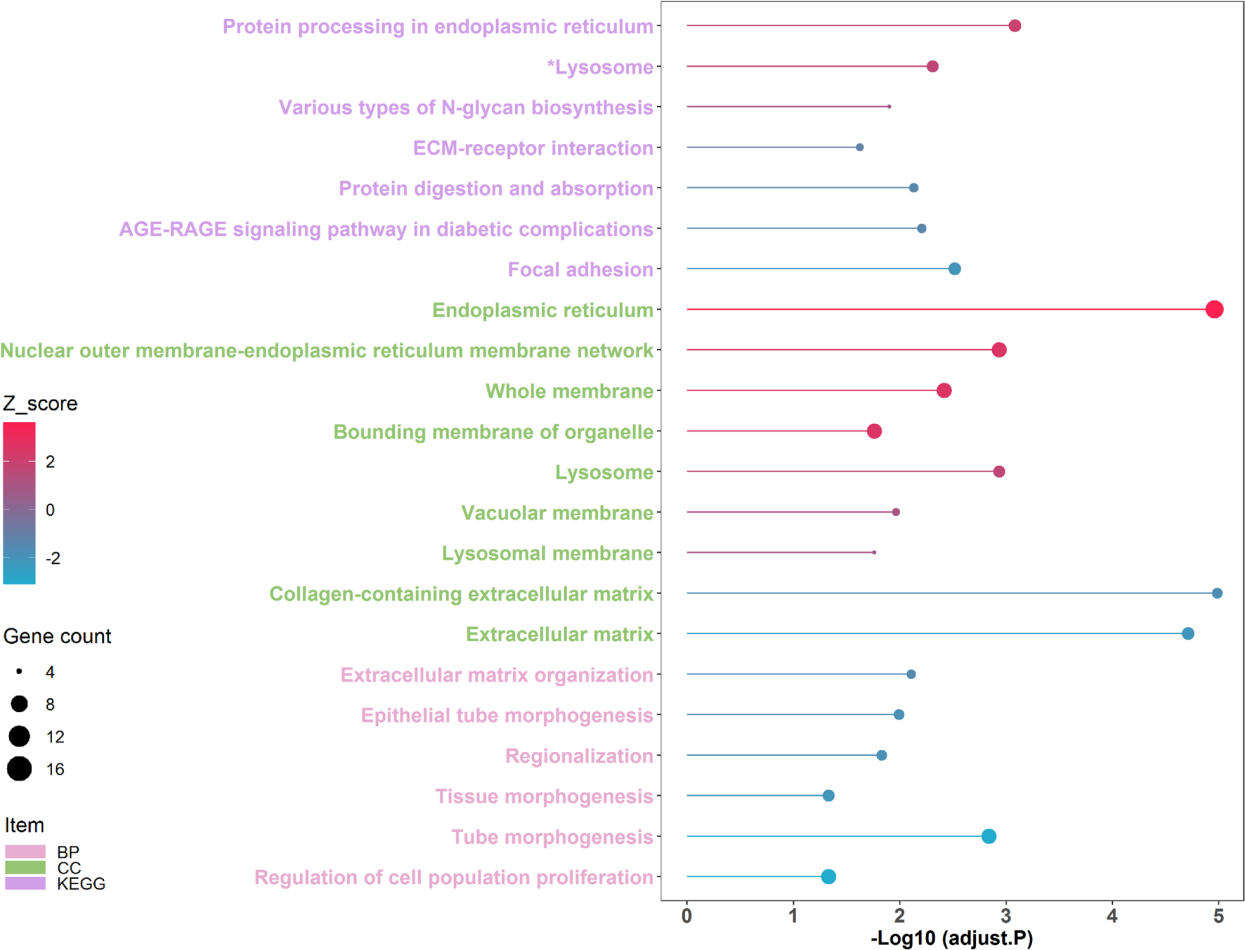
The functional enrichment analysis of the differentially expressed genes (DEGs)

**Fig. 3:**
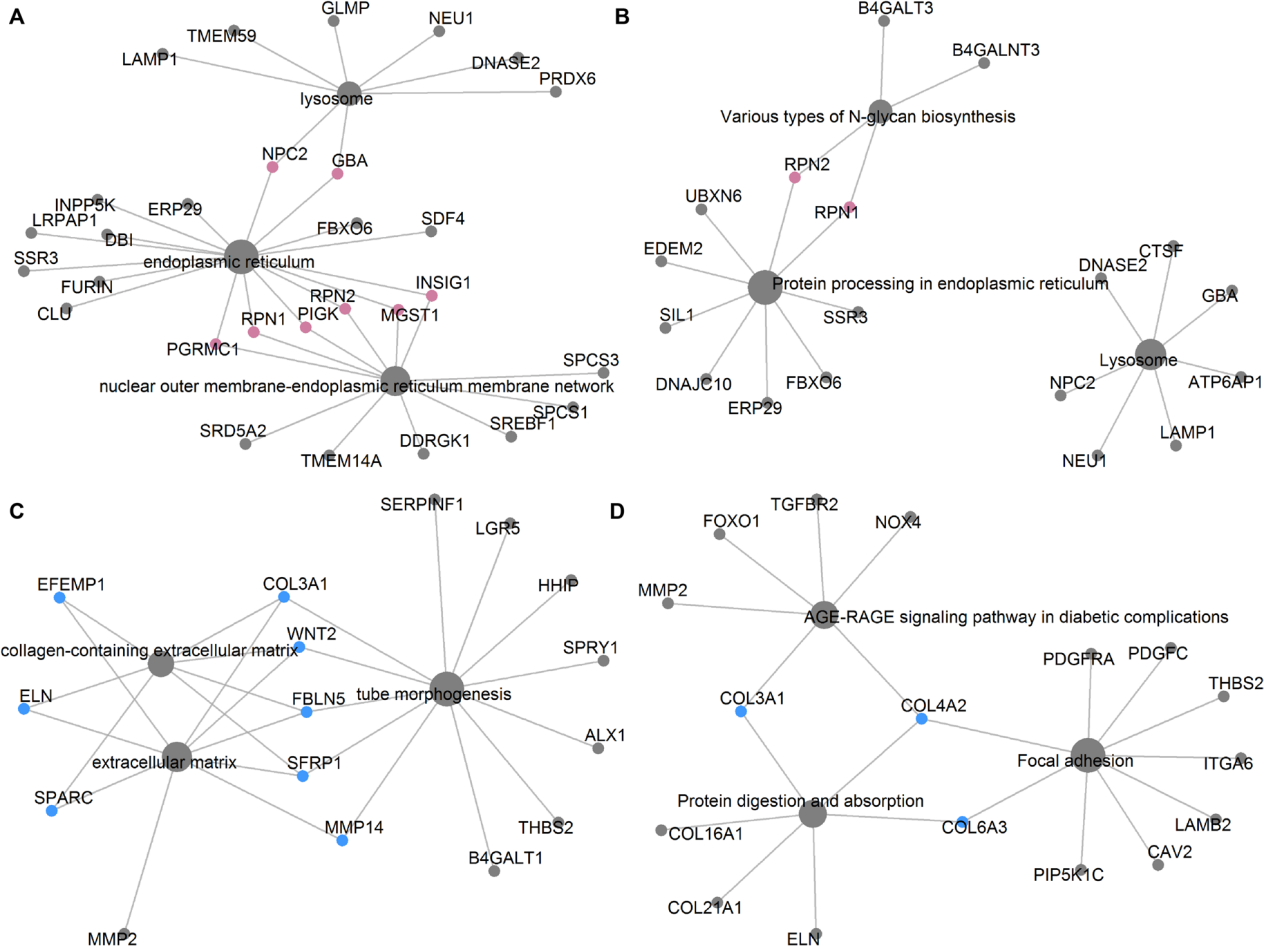
The node DEGs in the predominant GOs and KEGG functional categories. **A** The up-regulated node DEGs in the GOs functional categories. **B** The up-regulated node DEGs in the KEGG functional categories. **C** The down-regulated node DEGs in the GOs functional categories. **D** The down-regulated node DEGs in the KEGG functional categories

As shown in Fig. S3, a total of 361 differentially expressed genes (DEGs) were identified (FDR adjusted *P* < 0.01, Table S2), which including 198 gradually up-regulated DEGs and 163 gradually down-regulated DEGs along with postnatal development stages changes. Functional enrichment analysis showed that the up-regulated and down-regulated DEGs were enriched in 10 and 12 functional categories, respectively (FDR adjusted *P* < 0.05, Fig. 2, Table S3). Among these, endoplasmic reticulum, nuclear outer membrane-endoplasmic reticulum membrane network, protein processing in endoplasmic reticulum, lysosome, and various types of N-glycan biosynthesis were the dominant functional categories enriched by the enhanced DEGs corresponding to postnatal development stages changes, while the reduced DEGs mainly deposited into collagen-containing extracellular matrix, extracellular matrix, tube morphogenesis, focal adhesion, AGE-RAGE signaling pathway in diabetic complications, and protein digestion and absorption. To further understand the relationships between DEGs and predominant functional categories, the cnetplots were generated. In the up-regulated DEGs, 8 node genes *MGST1*, *RPN1*, *INSIG1*, *PGRMC1*, *PIGK*, *RPN2*, *NPC2*, and *GBA* had multiple connections with the dominant functional categories (Fig. 3A-B). In the down-regulated DEGs, 10 node genes *ELN*, *EFEMP1*, *SPARC*, *FBLN5*, *COL3A1*, *COL4A2*, *COL6A3*, *WNT2*, and *SFRP1* exhibited diverse connections with the outstanding functional categories (Fig. 3C-D).

### The features of porcine epididymal proteome at different postnatal developmental stages

**Fig. 4:**
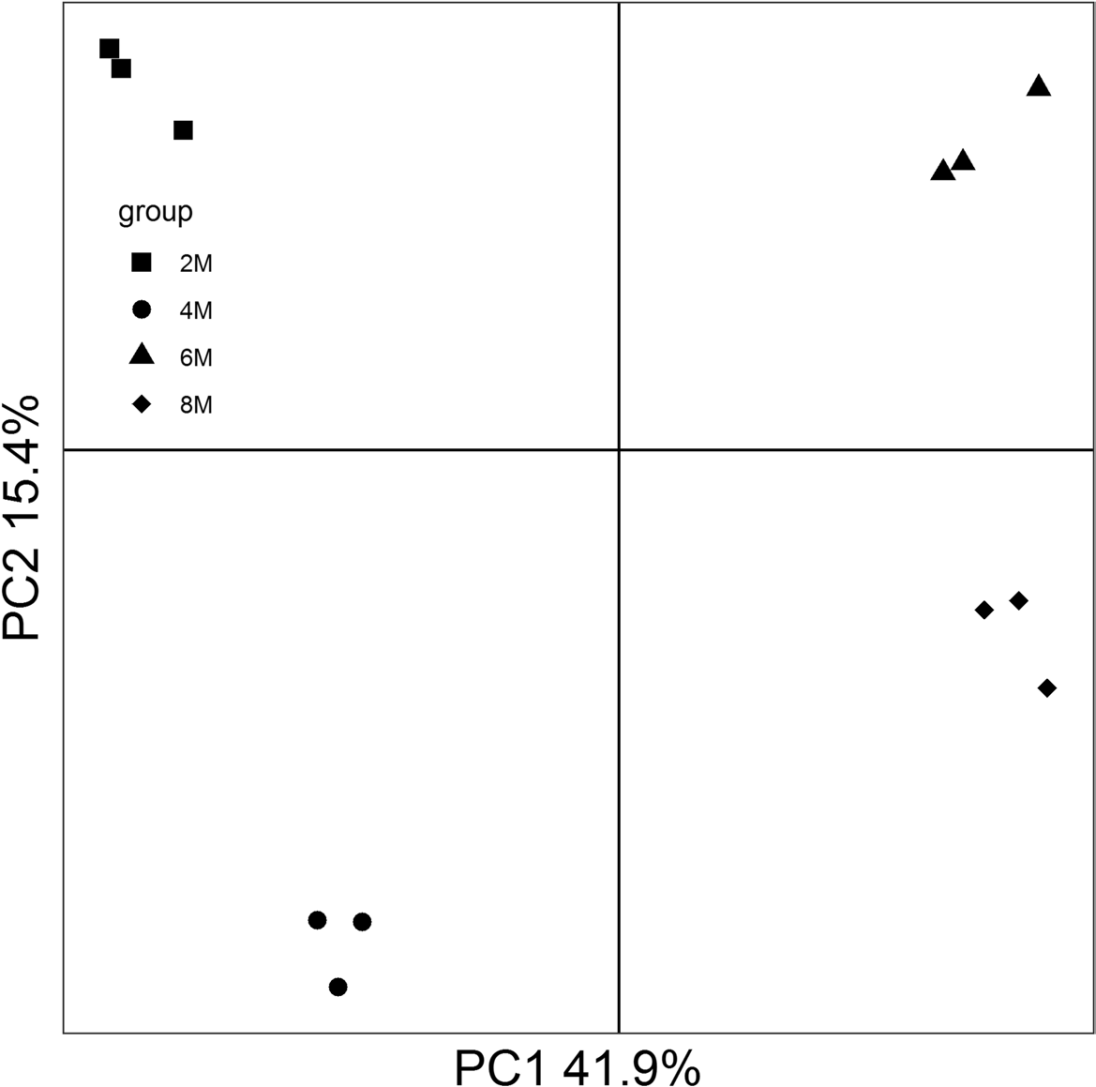
The PCA of porcine epididymal proteome at different postnatal development stages

Different from epididymal transcriptome sequencing results at different postnatal developmental stages, an almost same protein count was presented in 2-, 4-, 6-, and 8- month-old porcine epididymis (Fig. S4A). Moreover, 3,738 out of 3,739 proteins were shared by porcine epididymis at different postnatal developmental stages (Fig. S4B). Nonetheless, four clusters represent for porcine epididymal proteome at different developmental stages were clearly observed (Fig. 4).

Similar to the results of common genes functional annotation analysis, biological process, cellular component, molecular function, and KEGG pathways composed of 15, 2, 11, and 6 categories were annotated by common proteins (Fig. S5). Cellular process, metabolic process, biological regulation, localization, and response to stimulus were the dominant functional categories in the biological process. In the cellular component, common proteins annotated to cellular anatomical entity and protein-containing complex. Regarding the molecular function, binding, catalytic activity, molecular function regulator, structural molecule activity, and transporter activity were annotated by most common proteins. In addition, environmental information processing, human diseases, and metabolism were the predominant functional categories of the KEGG pathways.

### Unraveling postnatal development-dependent DEPs and functional items

**Fig. 5:**
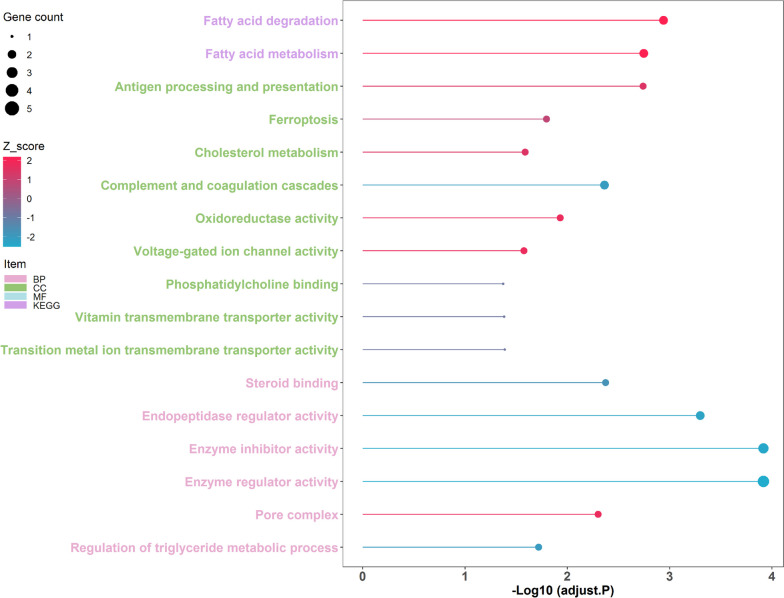
The functional enrichment analysis of the differentially expressed proteins (DEPs)

**Fig. 6:**
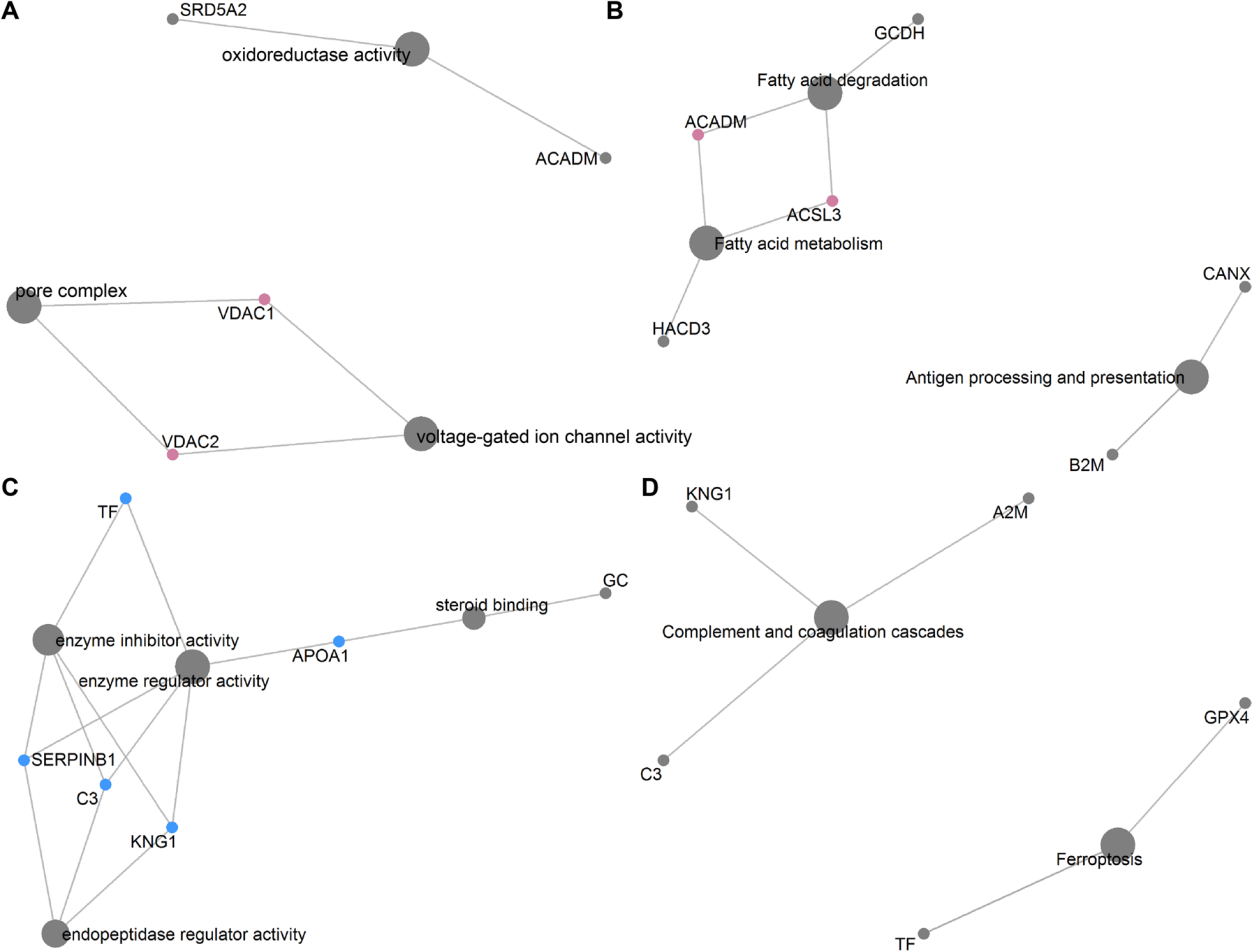
The node DEPs in the dominant GOs and KEGG functional categories. **A** The increased node DEPs in the GOs functional categories. **B** The increased node DEPs in the KEGG functional categories. **C** The reduced node DEPs in the GOs functional categories. **D** The reduced node DEPs in the KEGG functional categories

A total of 49 proteins showed significant alterations in abundances corresponding to postnatal developmental stage changes were unraveled (FDR adjusted *P* < 0.01, Fig. S6, Table S4). Of these, 25 proteins such as *SRD5A2*, *B2M*, *CANX*, *VDAC1*, and *VDAC2* showed increased expression levels during postnatal development and the remaining proteins including SERPINH1, APOA1, A2M, TF, and GPX4 exhibited decreased expression levels. The differentially expressed proteins (DEPs) were enriched in 18 functional items (FDR adjusted *P* < 0.05, Fig. 5, Table S5). The increased proteins mainly participated in fatty acid degradation, fatty acid metabolism, antigen processing and presentation, pore complex, oxidoreductase activity, and voltage-gated ion channel activity, while the decreased proteins mainly engaged in enzyme inhibitor activity, enzyme regulator activity, endopeptidase regulator activity, steroid binding, complement and coagulation cascades, and ferroptosis. Additionally, 9 node proteins were highlighted in the cnetplot (Fig. 6), which including 4 enhanced proteins VDAC1, VDAC2, ACADM, and ACSL3, and 5 reduced proteins KNG1, SERPINB1, C3, TF, and APOA1.

### Interaction network analysis and validation experiment

**Fig. 7:**
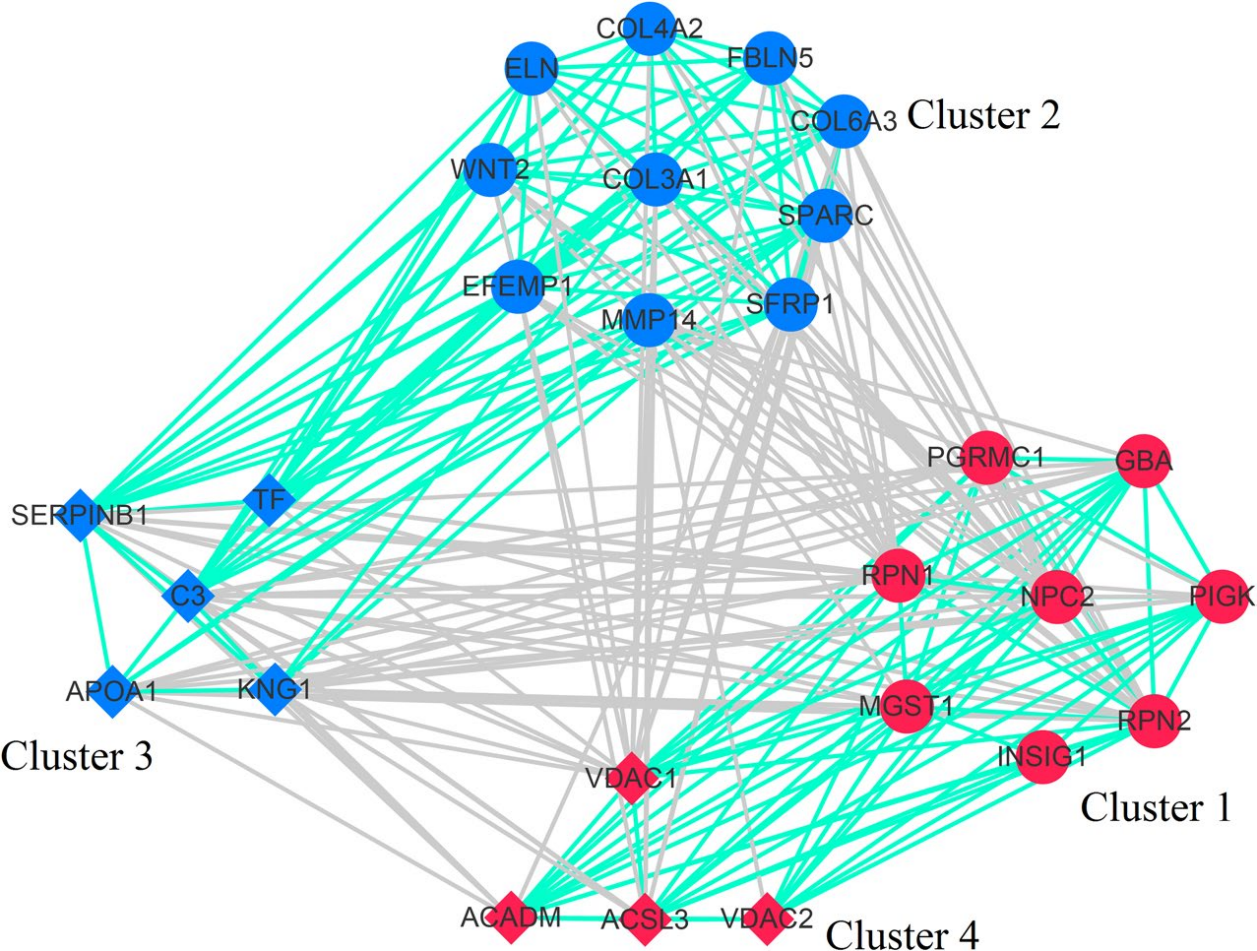
The interaction network of node DEGs and DEPs

**Fig. 8:**
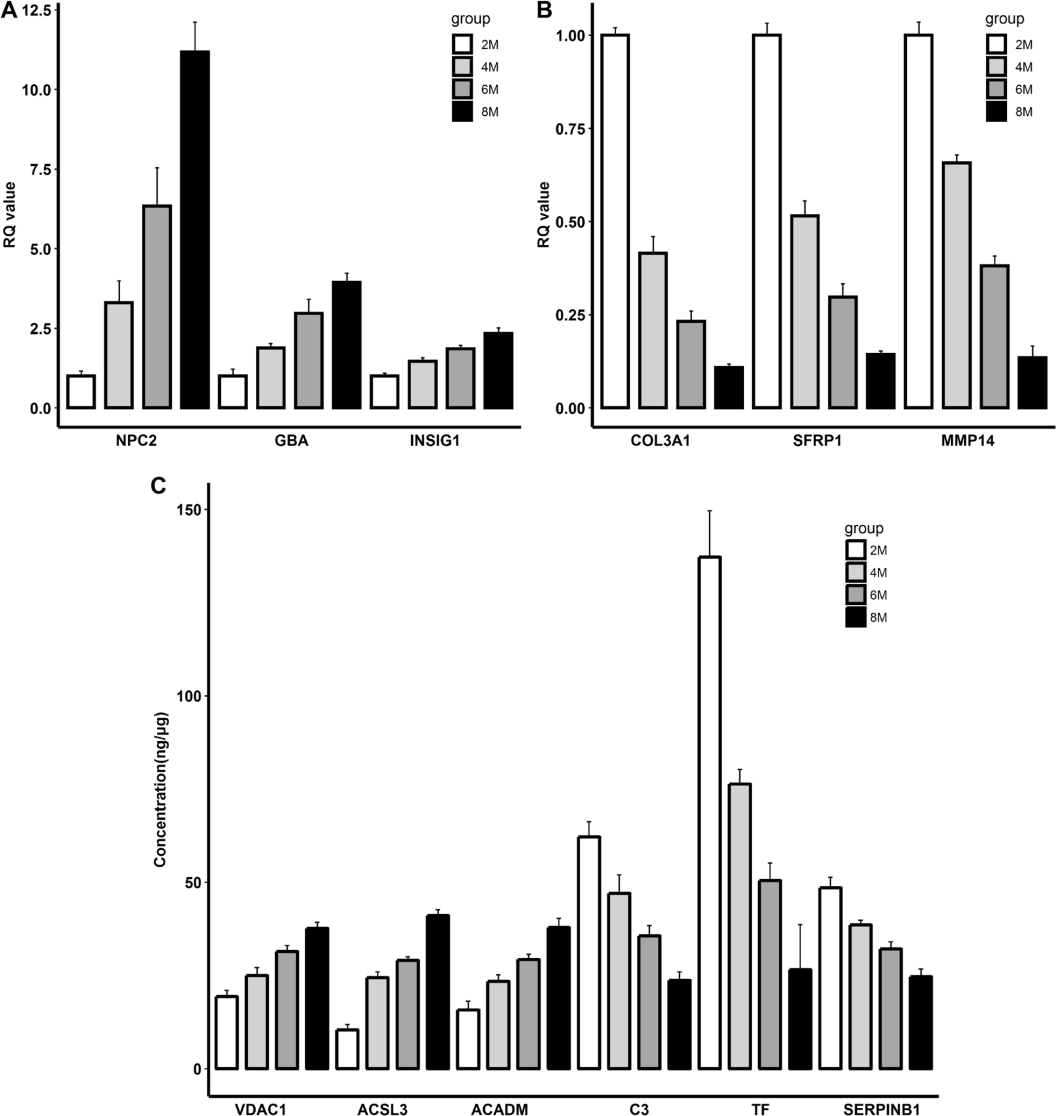
The validation experiments of DEGs (**A**-**B**) and DEPs (**C**)

To identify the hub genes and proteins, the spearman interaction network was constructed by using the above mentioned node genes and proteins. As shown in Fig. 7, four clusters represent for the differentially expressed genes and proteins were exhibited. The cluster 1 was formed by the increased expressed genes. The cluster 2 was comprised of the decreased expressed genes. The down- and up-regulated proteins constituted the cluster 3 and 4, respectively. Each component of the cluster had strong intra-interactions. Meanwhile, the cluster 1 showed inter-positive correlations with cluster 4, but showed inter-negative correlations with cluster 2 and 3. In contrast, the cluster 2 had inter-positive associations with cluster 3, but showed inter-negative associations with cluster 1 and 4. According to the maximal clique centrality, *NPC2*, *COL3A1*, C3, and VDAC1 were regarded as the hub nodes in the different clusters. Subsequently, the node genes and proteins possessed the top 3 maximal clique centrality in each cluster were verified by qRT-PCR and MRM, respectively. The result showed that these genes and proteins exhibited the expression patterns, which were observed in the transcriptome and proteome sequencing analysis (Fig. 8).

## Discussion

It is well-established that the epididymis produces a suitable environment for sperm maturation. Gaining integrative insights into the characteristics of epididymal genes and proteins expressions at different postnatal development stages is crucial for understanding the molecular basis of epididymal functions and offering some new insights into epididymal regulations. Nonetheless, it is still lacking in porcine epididymis studies. Hence, the alterations in both transcriptome and proteome of porcine epididymis at different postnatal developmental stages were determined in the present study.

The transcriptome sequencing results showed that the gene counts were increased during postnatal development and gene profiles were remarkably altered along with different development stages (Fig. S1 and Fig. 1). The similar results were observed in porcine testicular transcriptome analyses at different postnatal development stages [10], which implied that the transcription events of the gonad at the age of sexually mature were more active than those of the pubertal gonad. This result was further verified by the functional annotation analysis as the amounts of sexually mature stage specific genes assigned to the same functional items were generally greater than those of puberty stage specific genes (Fig. S2). It may be explained by the presences of stronger mRNA degradations in the pubertal epididymis [11].

We found that 198 and 163 DEGs were continuously up-regulated and down-regulated during postnatal development (Fig. S3). Functional enrichment analysis showed that most of the up-regulated DEGs such as *PGRMC1*, *RPN1*, *PIGK*, *RPN2*, *MGST1*, *INSIG1*, *NPC2*, and *GBA* linked to functions of endoplasmic reticulum and lysosome (Fig. 2). Similarly, previous studies have revealed that the activities of endoplasmic reticulum and lysosome of gonad cells are varied along with sexual maturity [8, 12]. The endoplasmic reticulum is the location of protein synthesis and assembly as well as lipid and membrane synthesis, which plays crucial roles in sperm maturation modulation [13, 14]. Meanwhile, the lysosome mediates the proper biochemical and molecular modifications of sperm by endocytosis and modifying glycoproteins and lipids on the sperm plasma membrane, which induces a fertile and motile state [15, 16]. Epididymal cholesterol homeostasis is vital for a proper post-testicular maturation of spermatozoa [17]. Interestingly, several key DEGs predominantly expressed in the endoplasmic reticulum and lysosome that may affect sperm maturation by modulating epididymal cholesterol metabolism (Fig. 3A-B). Insulin-induced gene 1 (*INSIG1*) is an essential endoplasmic reticulum membrane embedded sterol sensor that regulates cellular cholesterol homeostasis through a feedback inhibition mechanism [18]. Although progesterone receptor membrane component 1 (*PGRMC1*) is a member of a multi-protein progesterone-binding complex, it binds to the protein *INSIG1* engages in cholesterol homeostasis modulation as well [19]. Niemann-Pick C 2 (*NPC2*) is an intracellular cholesterol transporter that controls cholesterol export from lysosomes for cellular needs or storage [20]. Glucocerebrosidase (*GBA*) is a lysosomal retaining β-d-glucosidase catalyzes transglucosylation reaction to form β-cholesterylglucoside via the transfer of glucose residue from glucosylceramide to cholesterol, which inhibits the accumulation of cholesterol [21].

On the other hand, the down-regulated DEGs such as *SPARC*, *ELN*, *EFEMP1*, *COL3A1*, *WNT2*, and *MMP14* mainly related to properties of extracellular matrix. Consistently, the dynamic alterations of the gonadal extracellular matrix have been observed from puberty stage to sexual mature stage [22, 23]. Since the extracellular matrix is a reservoir for growth factors and bioactive molecules that orchestrate cell signaling, functions, properties, and morphology, it is naturally considered as a key player in modulating epididymal tissue homeostasis and sperm maturation [24, 25]. Some DEGs not only affect extracellular matrix construction but also associate with sperm physiological function were found (Fig. 3C-D). Both matrix metalloproteinase-2 (*MMP2*) and −14 (*MMP14*) are members of proteolytic enzymes family that participate in extracellular matrix remodeling by depredating protein components of the extracellular matrix [26]. Although the role of *MMP14* in sperm functionality is not well studied, the expression level of *MMP2* has been linked to the motility, morphology, and capacitation of bovine spermatozoa [27, 28]. Secreted frizzled-related protein 1 (*SFRP1*) involves in extracellular matrix assembly via activating matrix metalloproteinase [29]. The expression of *SFRP1* in adult rat testis is closely related to spermatid adhesion and sperm release [30]. Elastin (*ELN*) is a long-lived extracellular matrix protein, which exerts an important role in human sperm transport by regulating the contractile of testicular peritubular cells [31]. Wnt family member 2 (*WNT-2*) involves in maintaining the integrity of extracellular matrix components by interacting with extracellular matrix proteins [32]. The expression of *WNT-2* is found to be associated with chicken sperm motility [33]. Collagen type 3 alpha 1 chain (*COL3A1*) is associated with extracellular matrix receptor interaction signaling pathway that affect the formation efficiency of chicken spermatogonial stem cells in vitro [34]. Secreted protein acidic and rich in cysteine (*SPARC*) is a matricellular collagen-binding protein that implicated in extracellular matrix assembly, and has been reported to play a regulatory role in mouse germ cell maturation and differentiation [35, 36].

Divergent to transcriptome sequencing analysis, the proteomic profiling results indicated that an almost same amount of protein was present in the epididymis at different postnatal development stages (Fig. S4). Regulation processes of post-transcription (e.g., alternative splicing events, Fig. S7), translation, and protein degradation may lead to the difference, which emphasizes the importance of results obtained from the proteomic data for comprehensively understand the molecular basis of epididymal development and function [37]. Subsequently, 49 differentially expressed proteins corresponding to postnatal developmental stage changes were identified (Fig. S5). Previous studies have demonstrated that fatty acid compositions and mitochondrial membrane components changes parallel gonadal functional and structural development leading to germinal maturity [38, 39]. In this study, the up-regulated proteins were mainly engaged in fatty acid metabolism and mitochondrial membrane structural complex assembly such as pore complex and voltage-gated ion channel (Figs. 5 and 6). Fatty acid metabolism homeostasis is crucial for sperm membrane lipid composition remodeling during epididymal maturation, which ensures the function and integrity of spermatozoa [40]. Here, we observed two key differentially expressed proteins long-chain fatty acyl CoA synthetase 3 (ACSL3) and medium-chain acyl-CoA dehydrogenase (ACADM) related to fatty acid metabolism. ACSL3 is known to convert fatty acids to generate fatty acyl-coenzyme A esters that provide substrates for fatty acid β-oxidation, while ACADM is indispensable for the initiation of β-oxidation [41, 42]. The enhanced fatty acid β-oxidation is essential for sperm energy metabolism and maturation when spermatozoa pass and acquire motility in the epididymis [43]. Meanwhile, both voltage-dependent anion channel-1 (VDAC1) and −2 (VDAC2) are capable of forming stable and highly conductive voltage-gated channels, which provide the major passage for the transportation of ions and metabolites across the mitochondrial outer membrane [44]. Moreover, the abundances of VDAC1 and VDAC2 are closely related to spermatogenesis [45, 46].

It is well-known that the apoptotic and immune response processes exert vital roles in gonadal development and function maintenance during sexual maturity [47, 48]. Here, the key down-regulated proteins kininogen-1 (KNG1), complement component C3 (C3), transferrin (TF), and serpin family B member 1 (SERPINB1) are implicated in apoptotic and immune response processes. The reduction of pro-apoptotic protein KNG1 can promote the proliferation of endothelial cells, which is indispensable for the regulatory roles of epididymal epithelium in motility and fertilizing abilities acquisitions of spermatozoa [49, 50]. The inhibited activities of C3 and TF may have effects on immunosuppressive that protect the epididymal sperm against autoimmune responses and the establishment of immune tolerance [51, 52]. SERPINB1 has multiple modulatory roles in regulating the immune response and cellular homeostasis and the decreased abundance of SERPINB1 may be linked to the enhanced sex steroid hormone stimulus with the advancement of development [53, 54].

There are some limitations in the present study. One weakness is the small sample size. Collecting additional samples at each postnatal development stage will improve the statistical power and be beneficial to identify more DEGs and DEPs for illustrating the molecular alterations of epididymis during postnatal development. The validation experiment limited to several key DEGs and DEPs is another shortcoming. Verification of the other genes and proteins is required to confirm their roles in the molecular functions of epididymis. Additionally, enrolling the omics data derive from prenatal development stages will break through the limitation of our insight, which is essential for mapping the molecular landscape of epididymis.

## Conclusion

In this study, the transcriptome and proteome of porcine epididymis at different postnatal development stages were investigated. A total of 361 differentially expressed genes and 49 differentially expressed proteins corresponding to development stage changes were identified. Functional enrichment analysis indicated that most of DEGs linked to endoplasmic reticulum, lysosome, and extracellular matrix functions, while most of DEPs related to fatty acid metabolism, voltage-gated ion channel assembly, and apoptotic and immune processes. Our findings will facilitate advances in understanding of the molecular basis of porcine epididymis functions and give guidance for further mechanistic researches on epididymal sperm maturation. Of equal importance, our results should provide a basic reference for comprehensive understanding of mammalian epididymal activity at different postnatal development stages and emphasize on the necessity of using multi-omics methodology in solving issues of reproductive biology.

## Methods

### Animals and sample collection

Twelve healthy Duroc boars composed of each of three individuals at 2-, 4-, 6-, and 8-month of age were randomly selected from the pig farm of Yongcheng Animal Husbandry Technology Co., Ltd, Fuzhou, China. All pigs access to standard commercial diet and water *ad libitum*. After euthanasia by electrical stunning, epididymis tissue samples from different regions of each boar were collected and mixed. All samples were snap frozen in liquid nitrogen for transportation and stored at -80 °C until further RNA and protein extraction.

### RNA extraction and trancriptome sequencing analysis

The total RNA was extracted using RNeasy Midi kit (Qiagen, Germany) following the manufacturer’s protocol. The RNA integrity and concentration were checked by 2100 Bioanalyzer (Agilent Technologies, USA) and NanoDrop 2000 Spectrophotometer (Thermo Scientific, USA), respectively. The qualified RNA samples were sent to Biomarker Technologies Corporation (Beijing, China) for transcriptome sequencing analysis. In brief, cDNA library was prepared by using the NEB Next Ultra II RNA Library Prep Kit for Illumina (NEB, USA) according to the manufacturer’s instructions. The constructed library was sequenced on an Illumina HiSeq 4000 platform (Illumina Inc., USA) for 2 × 150 bp reads. The high quality data were obtained by removing the adapter sequences, ploy-N reads, and low-quality reads in raw data through the in-house Perl scripts. The clean reads were aligned to the reference genome *Sus scrofa* 11.1 by the HISAT2 (v2.0.4). StringTie (v1.3.4) was applied for transcriptome assembly. After gene expression level was quantified by Fragments per Kilobase per Million mapped reads (FPKM), one-way ANOVA analysis and multiple comparisons were conducted to identify differentially expressed genes (DEGs) corresponding to developmental stages changes with the criteria of false discovery rate (FDR) adjusted *P* < 0.01 using the R software (v4.0.5). The Gene Ontology (GO) and Kyoto Encyclopedia of Genes and Genomes (KEGG) [55] pathway enrichment analysis of DEGs were performed by using clusterProfiler R package.

### Protein extraction and iTRAQ labeling

The samples were ground to powder using liquid nitrogen, and subsequently sonicated in lysis buff (7 M Urea, 2 M thiourea and 3% CHAPS) containing protease inhibitor (1 mM PMSF). To obtain the supernatant, the samples were centrifuged at 140,000 g for 30 min at 4 °C. The concentrations of proteins were measured using the Pierce™ BCA protein assay kit (Thermo Scientific, USA). Before iTRAQ labeling, 100 µg of protein per sample was denatured and the cysteine residues were blocked, and then digested with 10 µg of sequencing-grade modified trypsin diluted in 0.5 M of tetraethylammonium bicarbonate (TEAB) buffer, and incubated at 37 °C overnight. The peptides were dried in a SpeedVac vacuum concentrator (Thermo scientific, USA) and dissolved in 0.5 M of TEAB. The peptides were labeled with the appropriate iTRAQ reagents following the instructions of the 8-plex iTRAQ labeling kit (AB SCIEX, USA) The iTRAQ-labeled peptides were mixed, aliquoted in 200 µg portions and dried by vacuum centrifugation. The dried iTRAQ-labeled peptides were sent to Biomarker Technologies Corporation (Beijing, China) for proteome analysis.

### LC-MS/MS analysis and data processing

The iTRAQ-labeled peptides were subjected to fractionation using strong cation-exchange chromatography (SCX) on a high-performance liquid chromatography (HPLC) system (Shimadzu, Kyoto, Japan) equipped with a silica-based SCX column (Ultremex column, 4.6 mm × 250 mm, Phenomenex, USA) prior to liquid chromatography/tandem mass spectrometry (LC-MS/MS) analysis. The LC-MS/MS analysis was performed on a Nano ACQUITY system (Waters Corporation, USA) coupled with an Orbitrap Fusion mass spectrometer (Thermo Scientific, USA). The peptides were firstly loaded onto a C18 trap column (Thermo Scientific, USA) and then eluted into a C18 analytical column (Thermo Scientific, 150 mm × 75 mm, 3 μm). Peptide elution was performed by using a linear gradient of solvents A (0.1% formic acid in water) and B (0.1% formic acid in acetonitrile) from 5 to 35% of solvent B in A at a constant flow rate of 300 nL/min over 90 min. After liquid-phase separation, the peptides were subjected to ionization follow by tandem mass spectrometry (MS/MS) analysis. The scans of peptide precursors from 350 to 1,600 m/z were performed at m/z 400 resolution with a 10^4^ ion count target. The 15 most abundant ions in each MS scan were automatically selected and fragmented in HCD mode to achieve high mass accuracy in the MS/MS spectra.

The raw data were processed by MaxQuant software (Martinsried, Germany), using the Andromeda search engine against the Uniprot-proteome_UP000008227-Susscrofa database. The parameters for the protein identification were set as follows: trypsin was specified as the digestion enzyme, cysteine carbamidomethylation as a fixed modification, protein N-terminal acetylation, oxidation of methionine, glutamine as pyroglutamic acid, and phosphorylation of tyrosine, serine and threonine were set as the variable modifications. 20 ppm was set as the mass deviation for fragments. The minimal peptide length was set to seven amino acids, and a maximum of two mis-cleavages was allowed. Confident protein identification is based on at least one unique peptide with FDR < 1.0%. Protein quantification was based on the intensity of the reported ions of the assigned peptides with at least two unique spectra. Differentially expressed proteins (DEPs) corresponding to development stages changes were identified by one-way ANOVA analysis and multiple comparisons with FDR adjusted *P* < 0.01. ClusterProfiler R package was used for GO and KEGG enrichment analysis of DEPs.

### Validation experiment of key genes and proteins

To validate the alternations in expression levels of the key genes among different groups, quantitative real-time PCR (qRT-PCR) was performed on ABI 7900 Fast Real-Time PCR System (Life, USA). 1 µg of total RNA isolated for trancriptome sequencing was subjected to reverse transcription using the PrimeScript RT Reagent Kit (Invitrogen, USA). 100 ng synthesized cDNA with the SYBR Green PCR Master Mix (Takara, Japan) in a final volume of 20 µL was used for qRT-PCR. The primer sequences were listed in Table S1. There were triplicate repeats for each sample. Cycle threshold (Ct) values were normalized to the internal reference gene β-actin and the changes in relative gene expression were analyzed by the 2^−ΔΔCt^ method.

Multiple reaction monitoring (MRM) assay was used to verify the changes in concentrations of key proteins according to the procedure described previously [56]. Briefly, at least one stable isotope-labeled standard (SIS) peptide for each target protein was synthesized (PTM Bio, China) based on the signal intensity and interference of the detected peptides to construct the calibration curves. 2 µg of digested peptides from each sample was analyzed on a TripleTOF 6600 System (AB SCIEX, Concord, USA) interfaced with a nano LC system (Shimadzu, Kyoto, Japan) to identify target peptides with significant MS/MS signals corresponding to the key proteins. The generated data were searched against the Uniprot database using ProteinPilot (AB SCIEX, USA). Then, the results were imported to Skyline software (v2.1) to establish the MRM transition list. All MRM samples from different groups were analyzed in triplicate using a QTRAP 6500 mass spectrometer (AB SCIEX, Framingham, USA) coupled with the nano LC 425 system (Eksigent, USA). The mobile phases consisted of solvent A (0.1% formic acid in water) and solvent B (98% acetonitrile with 0.1% formic acid). The flow rate was set at 300nL/min, and a 45 min water/acetonitrile gradient combined with a continuous increase of solvent B from 5 to 30% was used to separate the peptides. The obtained data was processed using Skyline software. MRM peak integrations were manually inspected to ensure correct peak detection and accurate integration. The concentration of each target protein was quantified based on the observed peak-area ratio.

### Statistical analysis

The Venn and principal component analysis (PCA) plot was generated using ggvenn and stats R packages, respectively. The functional annotation of common and specific genes and proteins was accomplished by searching against the online database David (https://david.ncifcrf.gov). The spearman correlation analysis was performed using R software to generate the correlation coefficient matrix. The correlation coefficient matrix was imported to igraph R package to identify the significant interactions (|r|> 0.8, FDR adjusted *P* < 0.05) among node genes and proteins. The interaction network was established using Cytoscape (v3.8.0). The hub genes and proteins were defined by the maximal clique centrality calculated using the cytoHubba plug-in.

### Supplementary Information

**Additional file 1. **

## Data Availability

The raw data support the study findings are available at China National GeneBank DataBase (https://db.cngb.org/) with accession number: CNP0004198.

## Abbreviations

DEGs: Differentially expressed genes
DEPs: Differentially expressed proteins
*AQP1*: Aquaporin-1
*SERPINA1*: Serpin family A member 1
FGF18: Fibroblast growth factor 18
*TLR2*: Toll-like receptor 2
*IL1A*: Interleukin 1 alpha
*CXCL10*: C-X-C motif chemokine 10
FDR: False discovery rate
GO: Gene Ontology
KEGG: Kyoto Encyclopedia of Genes and Genomes
qRT-PCR: Quantitative real-time PCR
PCA: Principal component analysis
INSIG1: Insulin-induced gene 1
*PGRMC1*: Progesterone receptor membrane component 1
*NPC2*: Niemann-Pick C 2
*GBA*: Glucocerebrosidase
*MMP-2*: Matrix metalloproteinase-2
*MMP-14*: Matrix metalloproteinase-14
*SFRP1*: Secreted frizzled-related protein 1
*ELN*: Elastin
*WNT-2*: Wnt family member 2
*COL3A1*: Collagen type 3 alpha 1 chain
*SPARC*: Secreted protein acidic and rich in cysteine
ACSL3: Long-chain fatty acyl CoA synthetase 3
ACADM: Medium-chain acyl-CoA dehydrogenase
VDAC1: Voltage-dependent anion channel-1
VDAC2: Voltage-dependent anion channel-2
KNG1: Kininogen-1
C3: Complement component C3
TF: Transferrin
SERPINB1: Serpin family B member 1

## References

[1] Park YJ, Kim JH, Kim HY, Park HB, Choe J, Kim GW (2020). The expression and localization of V-ATPase and cytokeratin 5 during postnatal development of the pig epididymis. Asian-Australas J Anim Sci.

[2] Hinton BT, Galdamez MM, Sutherland A, Bomgardner D, Xu B, Abdel-Fattah R (2011). How do you get six meters of epididymis inside a human scrotum?. J Androl.

[3] Browne JA, Yang R, Leir SH, Eggener SE, Harris A (2016). Expression profiles of human epididymis epithelial cells reveal the functional diversity of caput, corpus and cauda regions. Mol Hum Reprod.

[4] Jelinsky SA, Turner TT, Bang HJ, Finger JN, Solarz MK, Wilson E (2007). The rat epididymal transcriptome: comparison of segmental gene expression in the rat and mouse epididymides. Biol Reprod.

[5] Yuan H, Liu A, Zhang L, Zhou H, Wang Y, Zhang H (2006). Proteomic profiling of regionalized proteins in rat epididymis indicates consistency between specialized distribution and protein functions. J Proteome Res.

[6] Cyr DG, Hermo L, Robaire B (1993). Developmental changes in epithelial cadherin messenger ribonucleic acid and immunocytochemical localization of epithelial cadherin during postnatal epididymal development in the rat. Endocrinology.

[7] Wang H, Kumar TR (2012). Segment- and cell-specific expression of D-type cyclins in the postnatal mouse epididymis. Gene Expr Patterns.

[8] Aguilera AC, Carvelli L, Boschin V, Mohamed F, Zyla L, Sosa MA (2015). Changes in lysosomal enzymes and mannose-6-phosphate receptors related to sexual maturation in bull epididymis. Reprod Fertil Dev.

[9] Zhang GM, Lan S, Jia RX, Yan GY, Wang LZ, Nie HT (2016). Age-associated and tissue-specific expression of osteopontin in male Hu sheep reproductive tract. Tissue Cell.

[10] Ding H, Luo Y, Liu M, Huang J, Xu D (2016). Histological and transcriptome analyses of testes from Duroc and Meishan boars. Sci Rep..

[11] Zhang J, Liu Q, Zhang W, Li J, Li Z, Tang Z (2010). Comparative profiling of genes and miRNAs expressed in the newborn, young adult, and aged human epididymides. Acta Biochim Biophys Sin (Shanghai).

[12] Chen H, Chen K, Zhao F, Guo Y, Liang Y, Wang Z (2022). Macroautophagy involved in testosterone synthesis in Leydig cells of male dairy goat (Capra hircus). Theriogenology.

[13] Li Y, Zhao W, Fu R, Ma Z, Hu Y, Liu Y (2022). Endoplasmic reticulum stress increases exosome biogenesis and packaging relevant to sperm maturation in response to oxidative stress in obese mice. Reprod Biol Endocrinol.

[14] Guo W, Qu F, Xia L, Guo Q, Ying X, Ding Z (2007). Identification and characterization of ERp29 in rat spermatozoa during epididymal transit. Reproduction.

[15] Hermo L, Andonian S (2003). Regulation of sulfated glycoprotein-1 and cathepsin D expression in adult rat epididymis. J Androl.

[16] Carvelli L, Aguilera AC, Zyla L, Pereyra LL, Morales CR, Hermo L (2021). Castration causes an increase in lysosomal size and upregulation of cathepsin D expression in principal cells along with increased secretion of procathepsin D and prosaposin oligomers in adult rat epididymis. PLoS One.

[17] Saez F, Ouvrier A, Drevet JR (2011). Epididymis cholesterol homeostasis and sperm fertilizing ability. Asian J Androl.

[18] Ren R, Zhou X, He Y, Ke M, Wu J, Liu X (2015). PROTEIN STRUCTURE. Crystal structure of a mycobacterial Insig homolog provides insight into how these sensors monitor sterol levels. Science.

[19] Suchanek M, Radzikowska A, Thiele C (2005). Photo-leucine and photo-methionine allow identification of protein-protein interactions in living cells. Nat Methods.

[20] Oninla VO, Breiden B, Babalola JO, Sandhoff K (2014). Acid sphingomyelinase activity is regulated by membrane lipids and facilitates cholesterol transfer by NPC2. J Lipid Res.

[21] Akiyama H, Ide M, Nagatsuka Y, Sayano T, Nakanishi E, Uemura N (2020). Glucocerebrosidases catalyze a transgalactosylation reaction that yields a newly-identified brain sterol metabolite, galactosylated cholesterol. J Biol Chem.

[22] Chen H, Miao X, Xu J, Pu L, Li L, Han Y (2022). Alterations of mRNA and lncRNA profiles associated with the extracellular matrix and spermatogenesis in goats. Anim Biosci.

[23] Li T, Wang H, Luo R, An X, Li Q, Su M (2022). Proteome Informatics in Tibetan Sheep (Ovis aries) testes suggest the crucial proteins related to development and functionality. Front Vet Sci..

[24] Wong J, Damdimopoulos A, Damdimopoulou P, Gasperoni JG, Tran SC, Grommen SVH (2020). Transcriptome analysis of the epididymis from Plag1 deficient mice suggests dysregulation of sperm maturation and extracellular matrix genes. Dev Dyn.

[25] Yue B (2014). Biology of the extracellular matrix: an overview. J Glaucoma.

[26] Cabral-Pacheco GA, Garza-Veloz I, Castruita-De la Rosa C, Ramirez-Acuna JM, Perez-Romero BA, Guerrero-Rodriguez JF (2020). The roles of Matrix metalloproteinases and their inhibitors in Human Diseases. Int J Mol Sci..

[27] Warinrak C, Wu JT, Hsu WL, Liao JW, Chang SC, Cheng FP (2015). Expression of matrix metalloproteinases (MMP-2, MMP-9) and their inhibitors (TIMP-1, TIMP-2) in canine testis, epididymis and semen. Reprod Domest Anim.

[28] Kim SH, Song YS, Hwang SY, Min KS, Yoon JT (2013). Effects of hormones on the expression of matrix metalloproteinases and their inhibitors in bovine spermatozoa. Asian-Australas J Anim Sci.

[29] Foronjy R, Imai K, Shiomi T, Mercer B, Sklepkiewicz P, Thankachen J (2010). The divergent roles of secreted frizzled related protein-1 (SFRP1) in lung morphogenesis and Emphysema. Am J Pathol.

[30] Wong EW, Lee WM, Cheng CY (2013). Secreted frizzled-related protein 1 (sFRP1) regulates spermatid adhesion in the testis via dephosphorylation of focal adhesion kinase and the nectin-3 adhesion protein complex. FASEB J.

[31] Welter H, Herrmann C, Dellweg N, Missel A, Thanisch C, Urbanski HF (2020). The glucocorticoid receptor NR3C1 in testicular peritubular cells is developmentally regulated and linked to the smooth muscle-like Cellular phenotype. J Clin Med..

[32] Astudillo P, Larrain J (2014). Wnt signaling and cell-matrix adhesion. Curr Mol Med.

[33] Sun Y, Xue F, Li Y, Fu L, Bai H, Ma H (2019). Differences in semen quality, testicular histomorphology, fertility, reproductive hormone levels, and expression of candidate genes according to sperm motility in Beijing-You chickens. Poult Sci.

[34] Hu C, Zuo Q, Jin K, Zhao Z, Wu Y, Gao J (2022). Retinoic acid promotes formation of chicken (Gallus gallus) spermatogonial stem cells by regulating the ECM-receptor interaction signaling pathway. Gene..

[35] Riley HJ, Bradshaw AD (2020). The influence of the Extracellular Matrix in inflammation: findings from the SPARC-Null mouse. Anat Rec (Hoboken).

[36] Wilson MJ, Bowles J, Koopman P (2006). The matricellular protein SPARC is internalized in sertoli, Leydig, and germ cells during testis differentiation. Mol Reprod Dev.

[37] Wang P, Miao Y, Li XH, Zhang N, Wang Q, Yue W (2021). Proteome landscape and spatial map of mouse primordial germ cells. Sci China Life Sci.

[38] Wang H, Ding J, Ding S, Chang Y (2019). Metabolomic changes and polyunsaturated fatty acid biosynthesis during gonadal growth and development in the sea urchin Strongylocentrotus Intermedius. Comp Biochem Physiol Part D Genomics Proteomics..

[39] Garza S, Chen L, Galano M, Cheung G, Sottas C, Li L (2022). Mitochondrial dynamics, Leydig cell function, and age-related testosterone deficiency. FASEB J.

[40] Dambrova M, Cirule H, Svalbe B, Zvejniece L, Pugovichs O, Zorenko T (2008). Effect of inhibiting carnitine biosynthesis on male rat sexual performance. Physiol Behav.

[41] Zhao C, Liu G, Shang S, Wei Q, Zhang L, Xia T (2019). Adaptive evolution of the ACSL gene family in Carnivora. Genetica.

[42] Fukasawa M, Atsuzawa K, Mizutani K, Nakazawa A, Usuda N (2010). Immunohistochemical localization of mitochondrial fatty acid beta-oxidation enzymes in rat testis. J Histochem Cytochem.

[43] Shaia KL, Harris BS, Selter JH, Price TM. Reproductive functions of the mitochondrial progesterone receptor (PR-M). Reprod Sci. 2022;30(5):1443–52. 10.1007/s43032-022-01092-w 36255658

[44] Zinghirino F, Pappalardo XG, Messina A, Nicosia G, De Pinto V, Guarino F (2021). VDAC genes expression and regulation in mammals. Front Physiol.

[45] Amir A, Yanwirasti, Asmarinah, Oenzil F (2016). Alteration expression of Bax, Bcl-2 and VDAC1 genes in Oligozoospermic and fertile subjects. Pak J Biol Sci.

[46] Fang Y, Zhang L, Dong X, Wang H, He L, Zhong S (2020). Downregulation of vdac2 inhibits spermatogenesis via JNK and P53 signalling in mice exposed to cadmium. Toxicol Lett.

[47] Likszo P, Skarzynski DJ, Moza Jalali B (2021). Changes in Porcine Corpus Luteum Proteome Associated with Development, maintenance, regression, and rescue during estrous cycle and early pregnancy. Int J Mol Sci..

[48] Asadi A, Ghahremani R, Abdolmaleki A, Rajaei F (2021). Role of sperm apoptosis and oxidative stress in male infertility: a narrative review. Int J Reprod Biomed.

[49] Lalmanach G, Naudin C, Lecaille F, Fritz H (2010). Kininogens: more than cysteine protease inhibitors and kinin precursors. Biochimie.

[50] Chen H, Alves MBR, Belleannee C (2021). Contribution of epididymal epithelial cell functions to sperm epigenetic changes and the health of progeny. Hum Reprod Update.

[51] Kelly RW, Critchley HO (1997). Immunomodulation by human seminal plasma: a benefit for spermatozoon and pathogen?. Hum Reprod.

[52] Skibinski G, Kelly RW, Harrison CM, McMillan LA, James K (1992). Relative immunosuppressive activity of human seminal prostaglandins. J Reprod Immunol.

[53] Khillare GS, Sastry KVH, Agrawal R, Saxena R, Mohan J, Singh RP (2018). Expression of gonadotropin and sex steroid hormone receptor mRNA in the utero-vaginal junction containing sperm storage tubules of oviduct during sexual maturation in Japanese quail. Gen Comp Endocrinol.

[54] Singh LK, Pandey M, Baithalu RK, Fernandes A, Ali SA, Jaiswal L (2022). Comparative proteome profiling of Saliva between Estrus and Non-estrus stages by employing label-free quantitation (LFQ) and Tandem Mass Tag (TMT)-LC-MS/MS analysis: an Approach for Estrus Biomarker Identification in Bubalus bubalis. Front Genet..

[55] Kanehisa M, Furumichi M, Sato Y, Kawashima M, Ishiguro-Watanabe M (2023). KEGG for taxonomy-based analysis of pathways and genomes. Nucleic Acids Res.

[56] Wang K, Wang Y, Wang X, Ren Q, Han S, Ding L (2018). Comparative salivary proteomics analysis of children with and without dental caries using the iTRAQ/MRM approach. J Transl Med.

[57] Percie du Sert N, Ahluwalia A, Alam S, Avey MT, Baker M, Browne WJ (2020). Reporting animal research: explanation and elaboration for the ARRIVE guidelines 2.0. PLoS Biol..

