# Supplementary figures and images for: Identification of postnatal development dependent genes and proteins in porcine epididymis
Source: BMC Genomics. 2023 Dec 4;24:729. doi: 10.1186/s12864-023-09827-y (PMC10694963; doi:10.1186/s12864-023-09827-y)

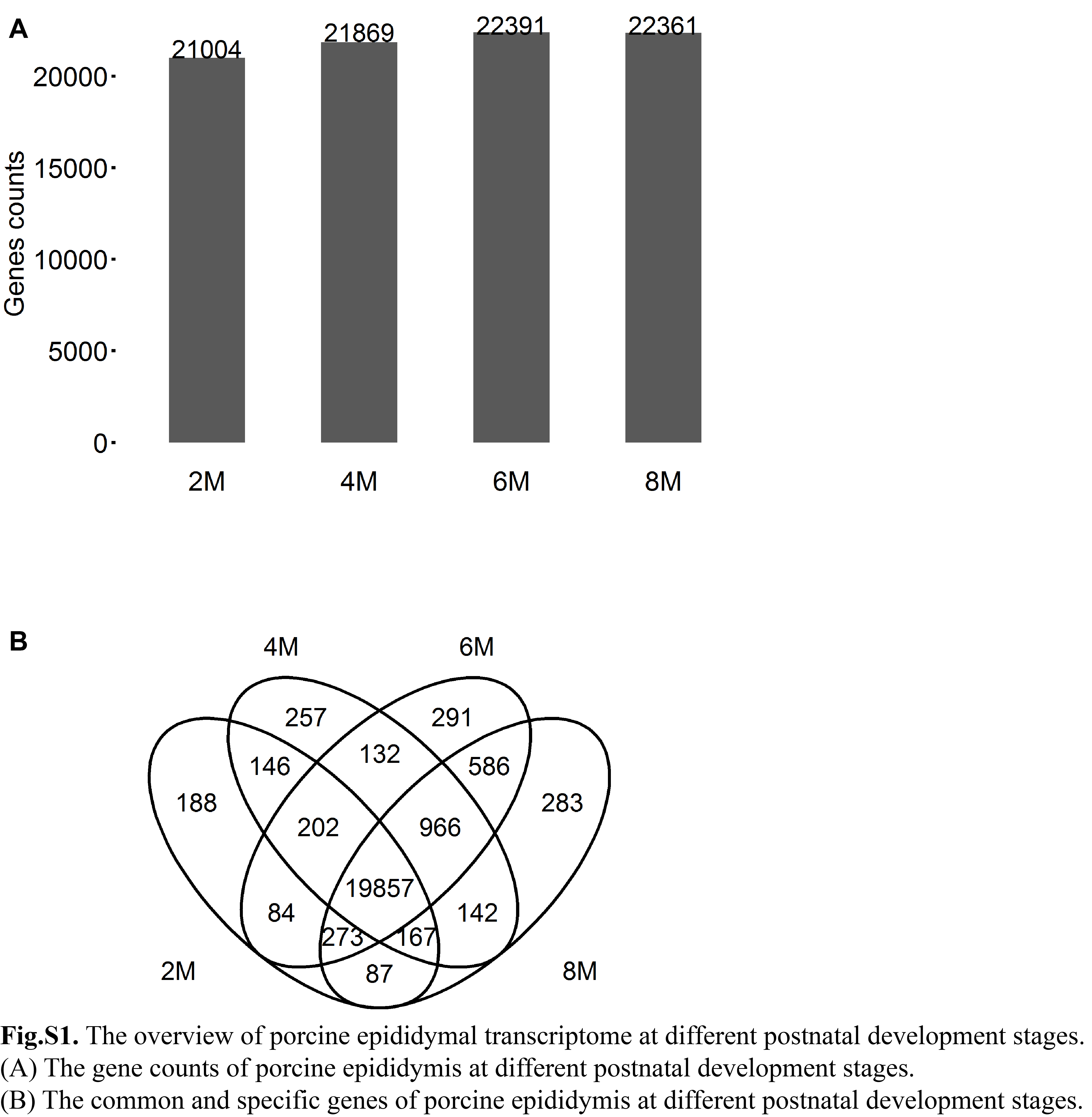

Supplement: Supplementary file 1 — Additional file 1. [file 12864_2023_9827_MOESM1_ESM.zip › Supplementary Files/Fig S1.tiff]

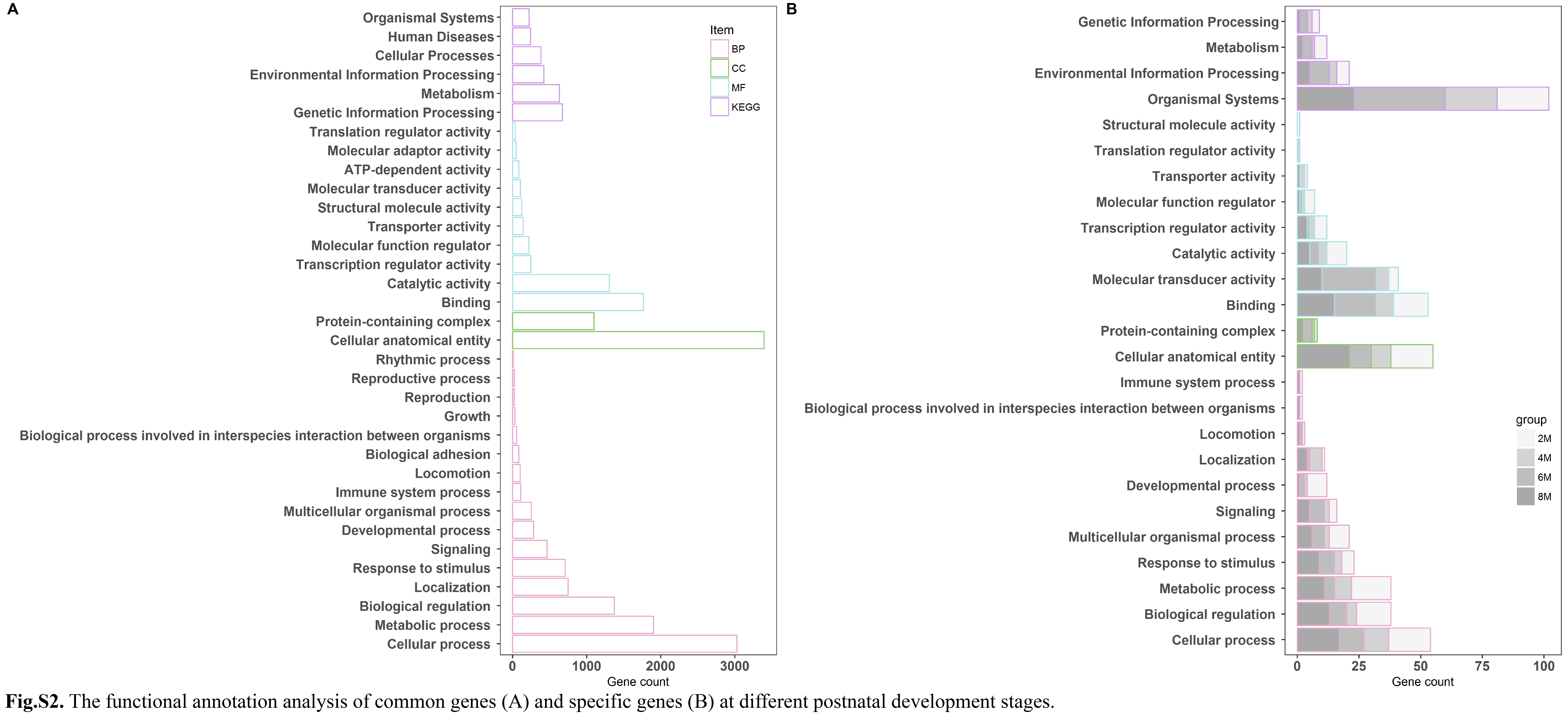

Supplement: Supplementary file 1 — Additional file 1. [file 12864_2023_9827_MOESM1_ESM.zip › Supplementary Files/Fig S2.tiff]

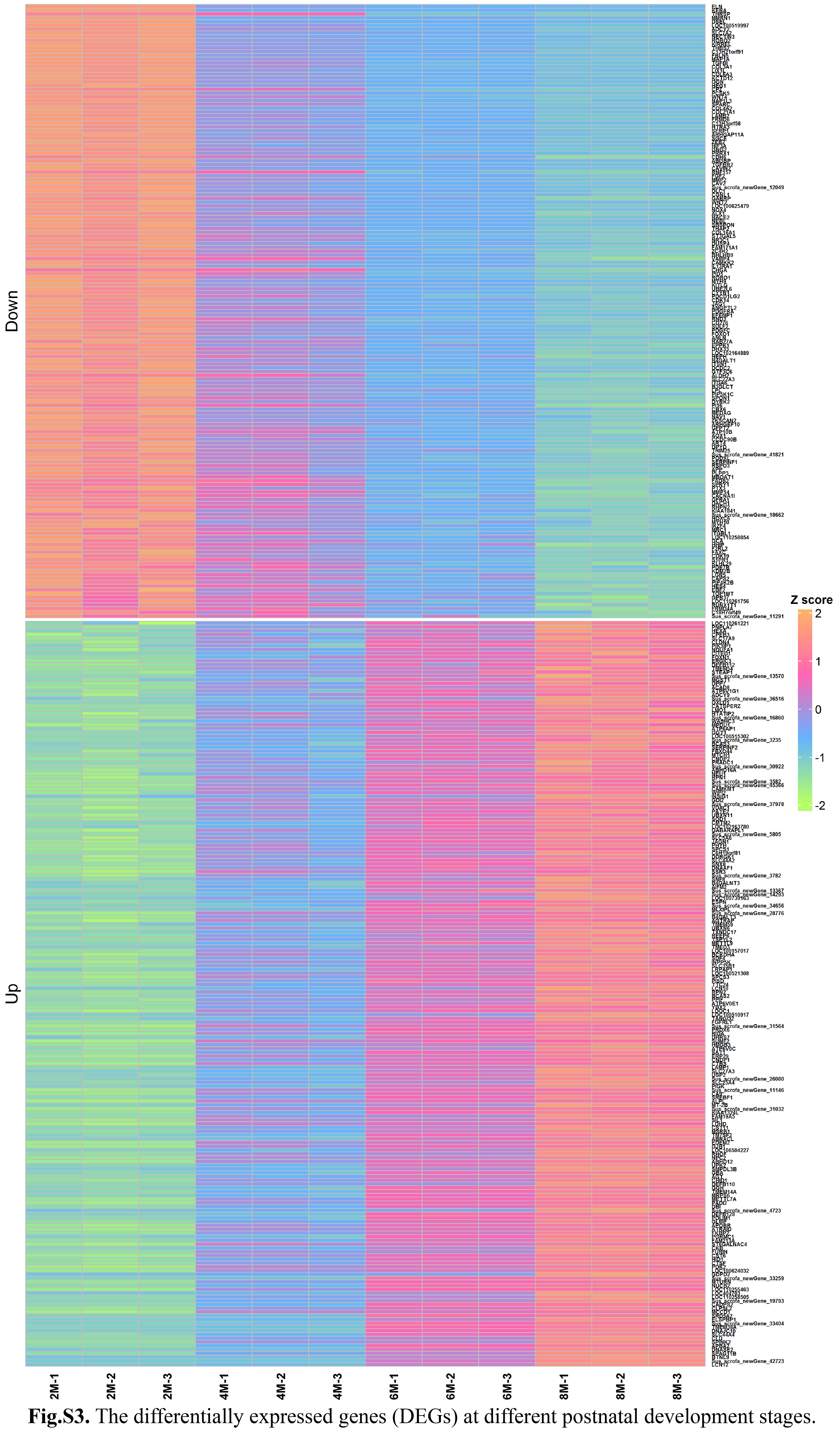

Supplement: Supplementary file 1 — Additional file 1. [file 12864_2023_9827_MOESM1_ESM.zip › Supplementary Files/Fig S3.tiff]

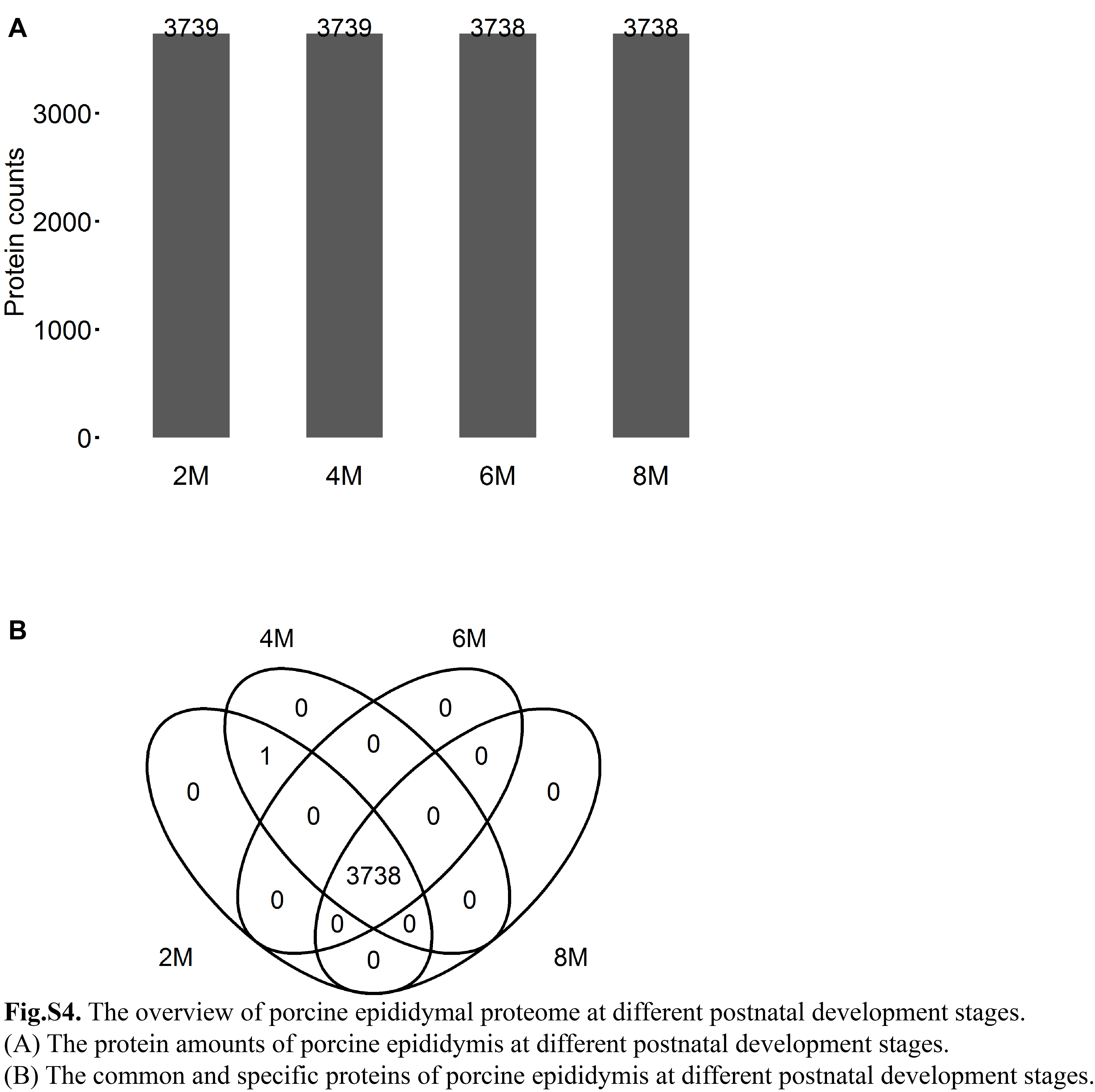

Supplement: Supplementary file 1 — Additional file 1. [file 12864_2023_9827_MOESM1_ESM.zip › Supplementary Files/Fig S4.tiff]

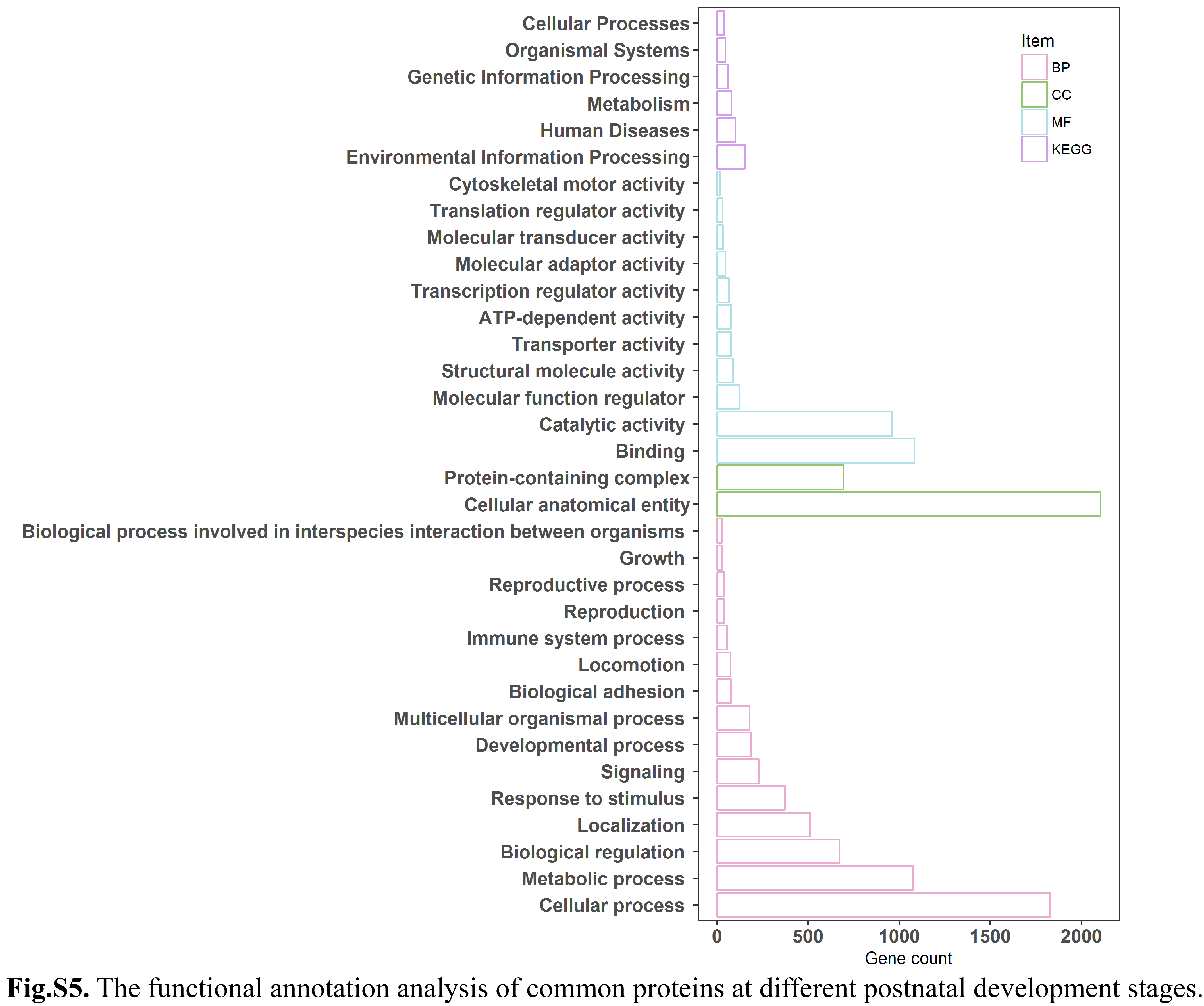

Supplement: Supplementary file 1 — Additional file 1. [file 12864_2023_9827_MOESM1_ESM.zip › Supplementary Files/Fig S5.tiff]

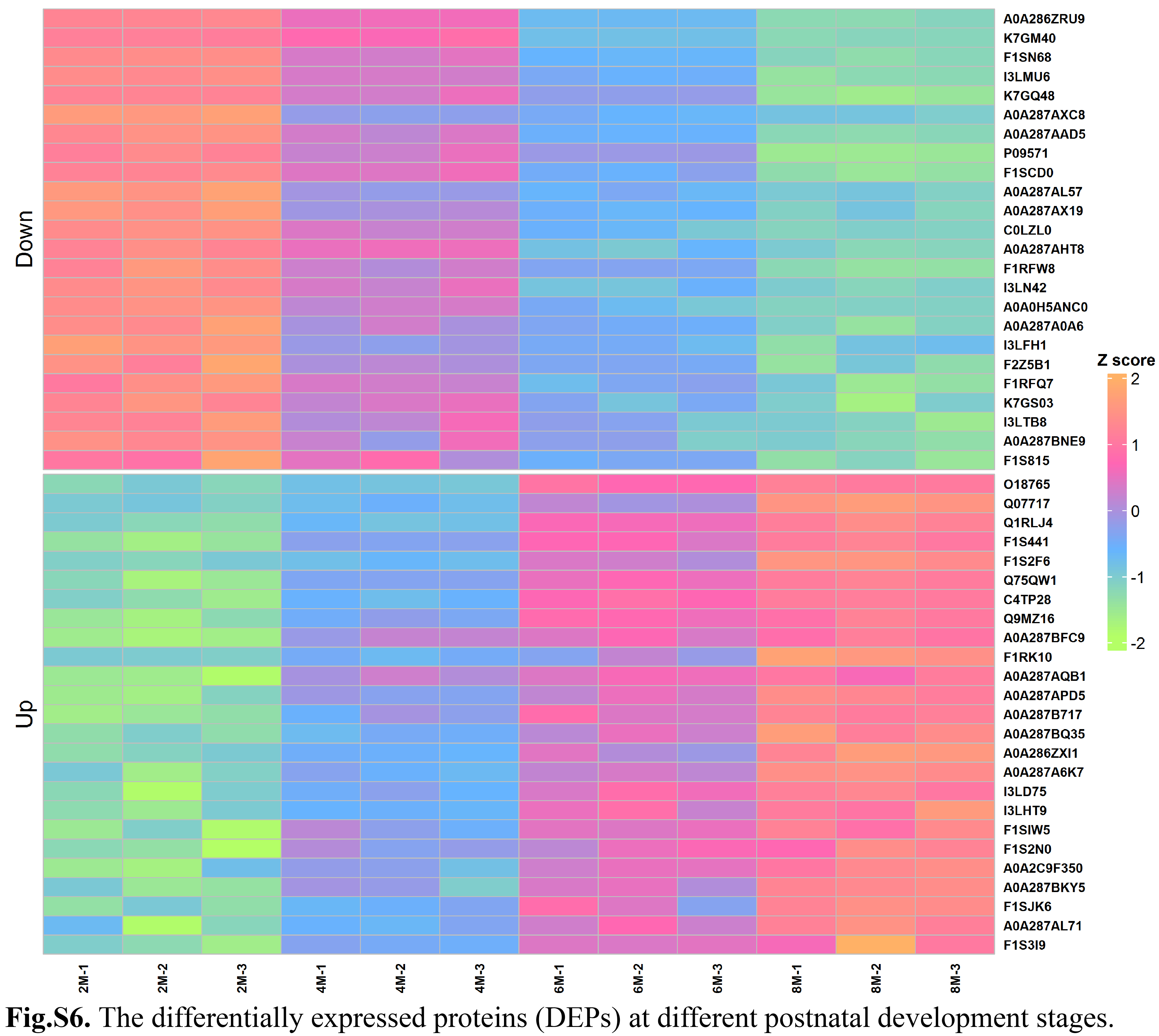

Supplement: Supplementary file 1 — Additional file 1. [file 12864_2023_9827_MOESM1_ESM.zip › Supplementary Files/Fig S6.tiff]

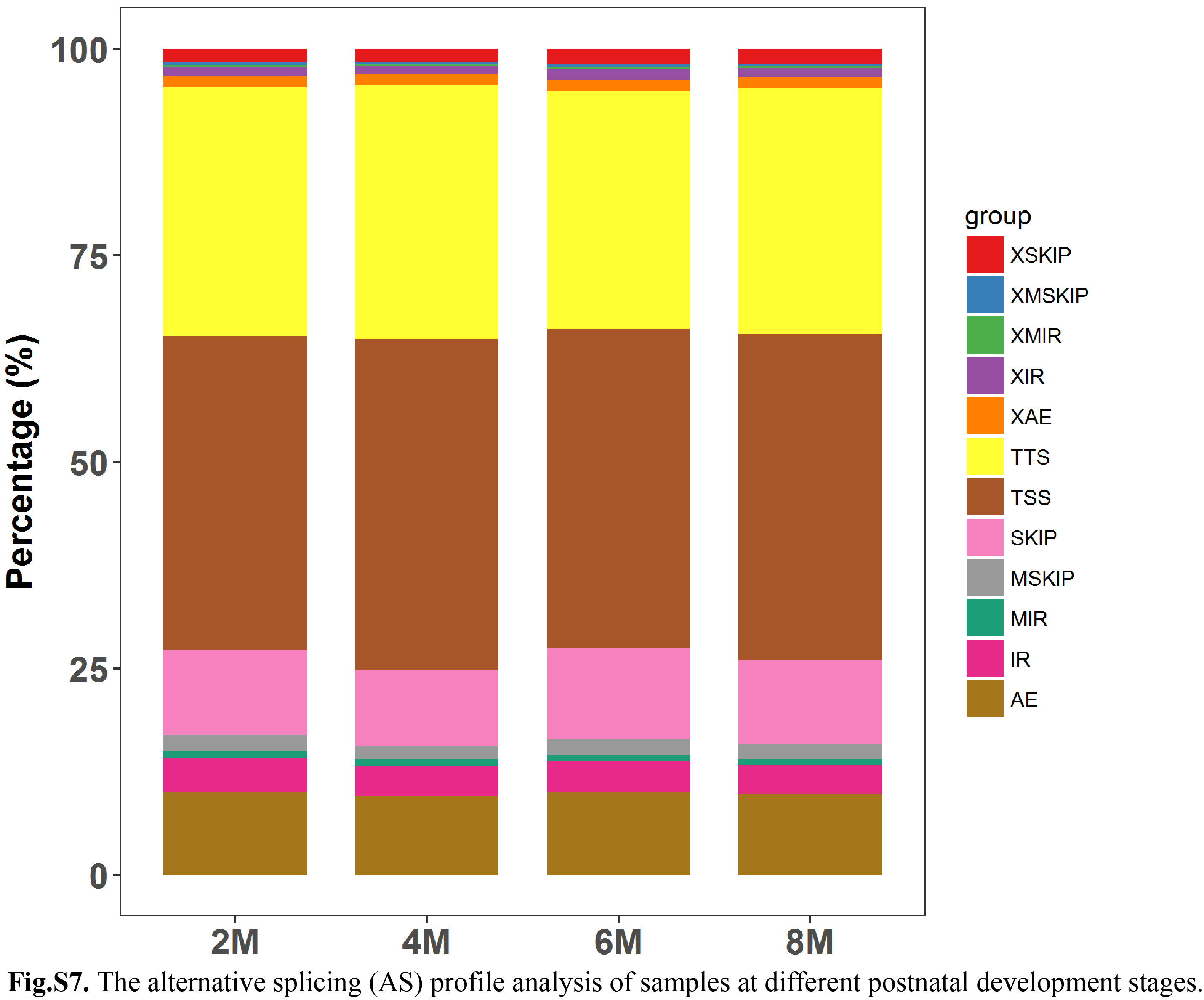

Supplement: Supplementary file 1 — Additional file 1. [file 12864_2023_9827_MOESM1_ESM.zip › Supplementary Files/Fig S7.tiff]
